# KnotProt: a database of proteins with knots and slipknots

**DOI:** 10.1093/nar/gku1059

**Published:** 2014-10-31

**Authors:** Michal Jamroz, Wanda Niemyska, Eric J. Rawdon, Andrzej Stasiak, Kenneth C. Millett, Piotr Sułkowski, Joanna I. Sulkowska

**Affiliations:** 1 Faculty of Chemistry, University of Warsaw, Pasteura 1, 02-093 Warsaw, Poland; 2 Institute of Mathematics, University of Silesia, Bankowa 14, 40-007 Katowice, Poland; 3 Department of Mathematics, University of St. Thomas, Saint Paul, MN 55105, USA; 4 Center for Integrative Genomics, University of Lausanne, 1015-Lausanne, Switzerland; 5 Department of Mathematics, University of California, Santa Barbara, CA 93106, USA; 6 Faculty of Physics, University of Warsaw, Pasteura 5, 02-093 Warsaw, Poland; 7 California Institute of Technology, Pasadena, CA 91125, USA; 8 Centre of New Technologies, University of Warsaw, Banacha 2c, 02-097, Warsaw, Poland

## Abstract

The protein topology database KnotProt, http://knotprot.cent.uw.edu.pl/, collects information about protein structures with open polypeptide chains forming knots or slipknots. The knotting complexity of the cataloged proteins is presented in the form of a matrix diagram that shows users the knot type of the entire polypeptide chain and of each of its subchains. The pattern visible in the matrix gives the knotting fingerprint of a given protein and permits users to determine, for example, the minimal length of the knotted regions (knot's core size) or the depth of a knot, i.e. how many amino acids can be removed from either end of the cataloged protein structure before converting it from a knot to a different type of knot. In addition, the database presents extensive information about the biological functions, families and fold types of proteins with non-trivial knotting. As an additional feature, the KnotProt database enables users to submit protein or polymer chains and generate their knotting fingerprints.

## INTRODUCTION

The KnotProt database, http://knotprot.cent.uw.edu.pl/, presents information about proteins with slipknots and knots. This is the first database that classifies proteins with slipknots and knots, represents their entire complexity in the form of a ‘knotting fingerprint’ (1) and presents many biological and geometrical statistics based on these data. The KnotProt database is based on protein chains deposited in the Protein Data Bank (PDB).

Currently, there are over 100 000 structures deposited in the PDB. The first examples of knots in proteins were proposed in (2,3), the first deep knot was identified in (4) and, since then, many more have been identified (1,5–7). Proteins may also form slipknots, i.e. contain knotted subchains even though their backbone chain as a whole is unknotted (1); they were discovered in proteins in 2007 (8) and their systematic analysis was initiated in 2009 (9). Usually it is impossible to determine, by a naked eye, if a given protein chain forms a knot or a slipknot. Therefore, more involved mathematical tools, such as polynomial knot invariants, are used to detect chain knotting and slipknotting. Much effort has been invested into identifying knotted proteins among those deposited in the PDB.

Recently considerable interest arose around this subject for a variety of reasons, both from a theoretical (10–19) and an experimental (20–24) perspective. First, it is believed that the presence of knots and slipknots in proteins is not accidental and therefore understanding their function is an important challenge. Second, recent work shows nearly perfect conservation of knotting fingerprints in some families whose members differ by hundreds of millions years of evolution (arising from distant organisms) and possess a low sequence identity (1). Moreover, based on knotting fingerprints, it was shown that the locations of active sites in proteins (based on ubiquitin C-terminal hydrolases proteins and five families of proteins with knotting notation S3_1_4_1_3_1_) are correlated with points characterizing their topology (e.g. positions of the knot core) (1). These findings imply that a detailed representation of protein topology can be crucial for understanding their biological role. The KnotProt database will make knotting and slipknotting data easily available and should help researchers to understand biological reasons of protein knotting.

**Figure 1.**
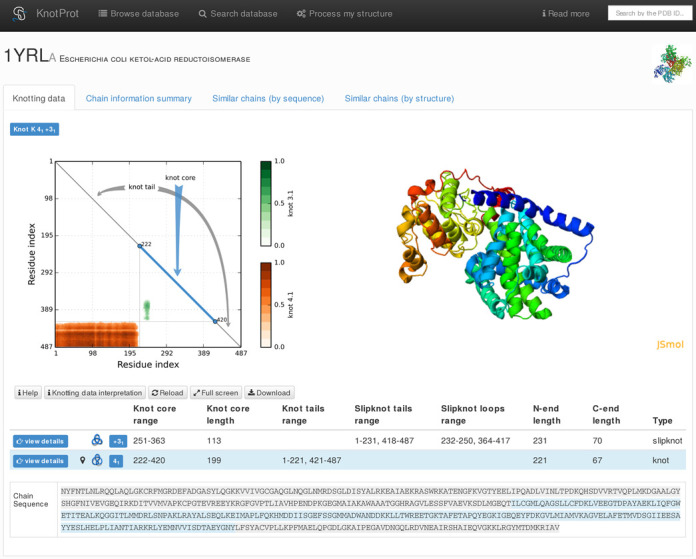
An example of data presentation for a knotted protein (PDB code 1yrl) in the KnotProt database. In this example the analyzed polypeptide chain of *Escherichia coli* ketol-acid reductoisomerase reveals that the entire polypeptide chain forms a 4_1_ knot, and has a subchain forming a 3_1_ knot. Diagram in top left: knotting fingerprint revealing the positions of subchains forming 4_1_ and 3_1_ knots. Top right: graphical representation of protein structure in JSmol. Table in the middle: detailed data about knots and slipknots formed by backbone subchains. Bottom: sequence representation with the knot core and knot tails highlighted in appropriate colors.

The KnotProt database contains detailed information about the entanglement in proteins and presents it in the form of a ‘knotting fingerprint.’ The knotting fingerprint encodes information about the knot type of each subchain of a protein backbone and represents it in the form of a matrix diagram, see Figure 1 and the detailed description in the ‘MATERIALS AND METHODS’ section. The KnotProt database also presents extensive statistics about proteins with knots and slipknots based on their biological function, molecular tags, family association, type of fold, as well as geometric data: knotting patterns, knot and slipknot lengths and depths, etc. Interestingly, the KnotProt analysis reveals that proteins with knots and slipknots can be classified into a few distinct motifs, represented by particular patterns within the matrix diagrams. These data can be used, for example, to find proteins with knots or slipknots with a given homological sequence, a similar structure, or performing a particular biological function. As an additional feature, a user can analyze structures and generate knotting fingerprints of uploaded proteins. It is also possible to upload and analyze a whole set of structures (e.g. analyze the evolution of a knot along a folding or unfolding trajectory). The KnotProt database is automatically updated every week, immediately after new structures are deposited in the PDB.

## MATERIALS AND METHODS

### Knot detection

**Figure 2.**
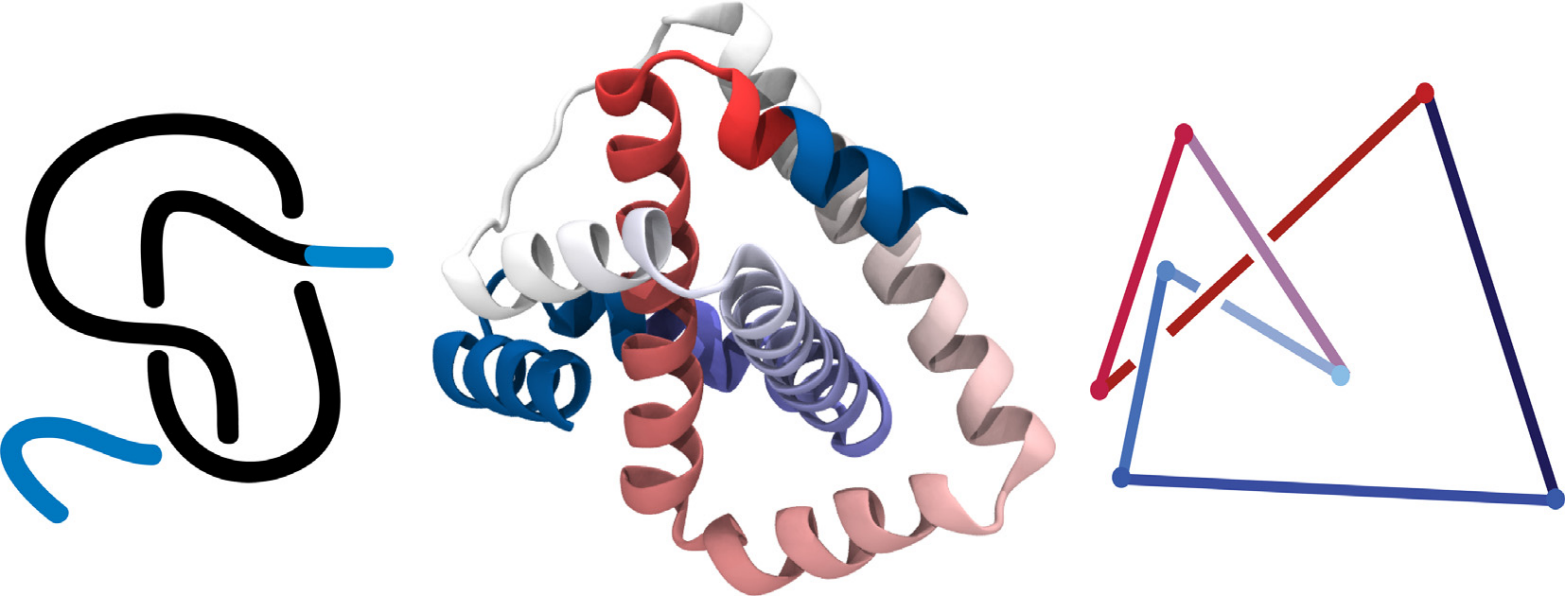
A protein with a trefoil (3_1_) knot (middle panel). Left panel: schematic representation of trefoil (3_1_) knot in an open chain. Right panel: simplified representation of the backbone chain of the protein in the middle panel, obtained after chain closure and simplification by the KMT algorithm (see the main text). The KnotProt uses such simplified polygonal configurations to calculate knot polynomials.

An example of a knotted protein is shown in the middle panel of Figure 2. Even this simple example requires a trained eye to realize that it is entangled and contains a trefoil knot, shown schematically in the left panel. In order to detect knots we analyze polygonal line segments with coordinates of vertices corresponding to the locations of the alpha carbons in the sequential amino acids of the polypeptide chains of the analyzed protein structures. After connecting the two termini of a protein and reducing the structure (in a way which preserves its topological type) we obtain a simplified polygonal configuration, as shown in the right panel of Figure 2. In what follows we discuss how KnotProt detects knots and constructs knotting fingerprints.

Knots are the basic objects studied in the mathematical field of knot theory. Knot theory studies entanglement in closed chains, although the ideas can be extended to characterize knotting in open chains (which we describe more below) (25). Several types of knots have been found so far in proteins. These are known and denoted as follows: trefoil (denoted also as 3_1_), figure-8 (denoted 4_1_), 5_2_ and Stevedore's knot (denoted 6_1_). An unknotted loop is called the trivial knot, or the unknot and is denoted 0_1_. The knots mentioned above are presented in the screenshot in Figure 5. In the notation above, the first number denotes the minimal number of crossings a given knot can show in a projection (e.g. minimal number of crossings in a projection of a trefoil onto a plane is 3). The 3_1_, 5_2_ and 6_1_ knots are chiral, i.e. they differ from their mirror images, and their complete characterization requires the determination of their chirality, which we denote by a plus (+) or a minus (−) sign next to the symbol of a knot. The 4_1_ knot is an example of an achiral knot, i.e. it is identical to its mirror image and cannot be assigned a chirality.

**Figure 3.**
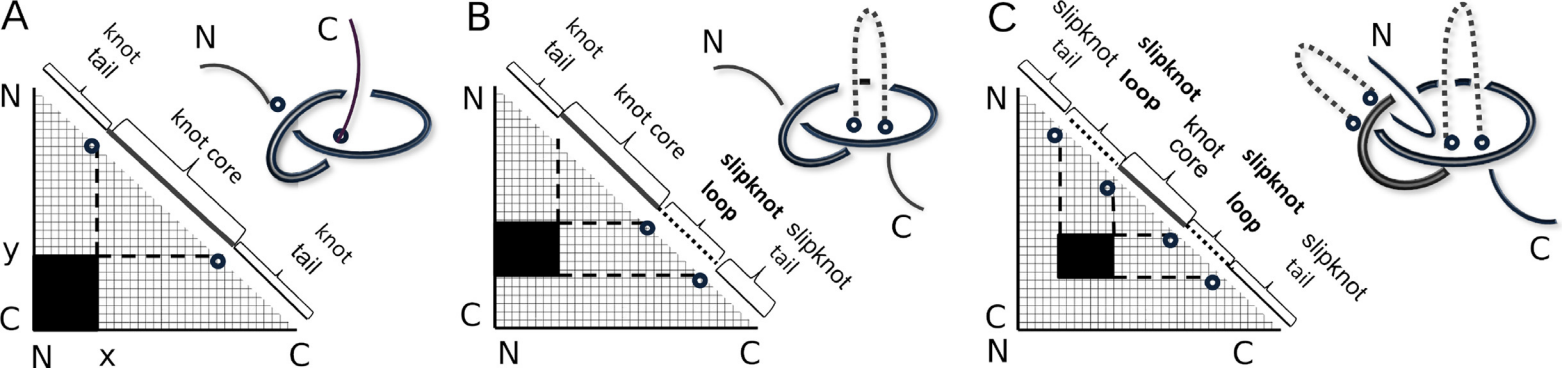
Examples of knotting fingerprints (figure from (1)) for a knot (A) and two slipknots (B and C). For a knot (panel A) the shaded area necessarily includes a point in the left-bottom corner of the diagram. For slipknots (B and C) this point is not included in the shaded area.

**Figure 4.**
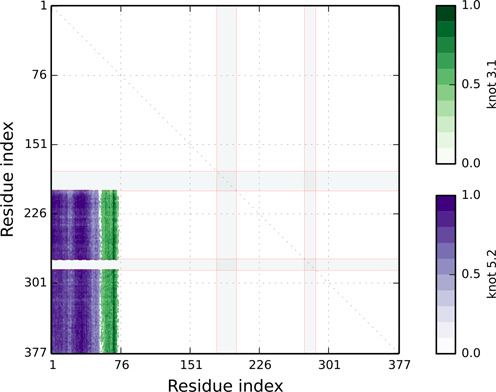
Missing atoms are denoted by gray strips in the knotting fingerprint, example based on protein 2cav. If a PDB structure contains missing atoms, its knotting type may depend on the space configuration of the missing segment and the knotting type of the chain may not be properly detected in the KnotProt—a user should be careful when interpreting such results.

**Figure 5.**
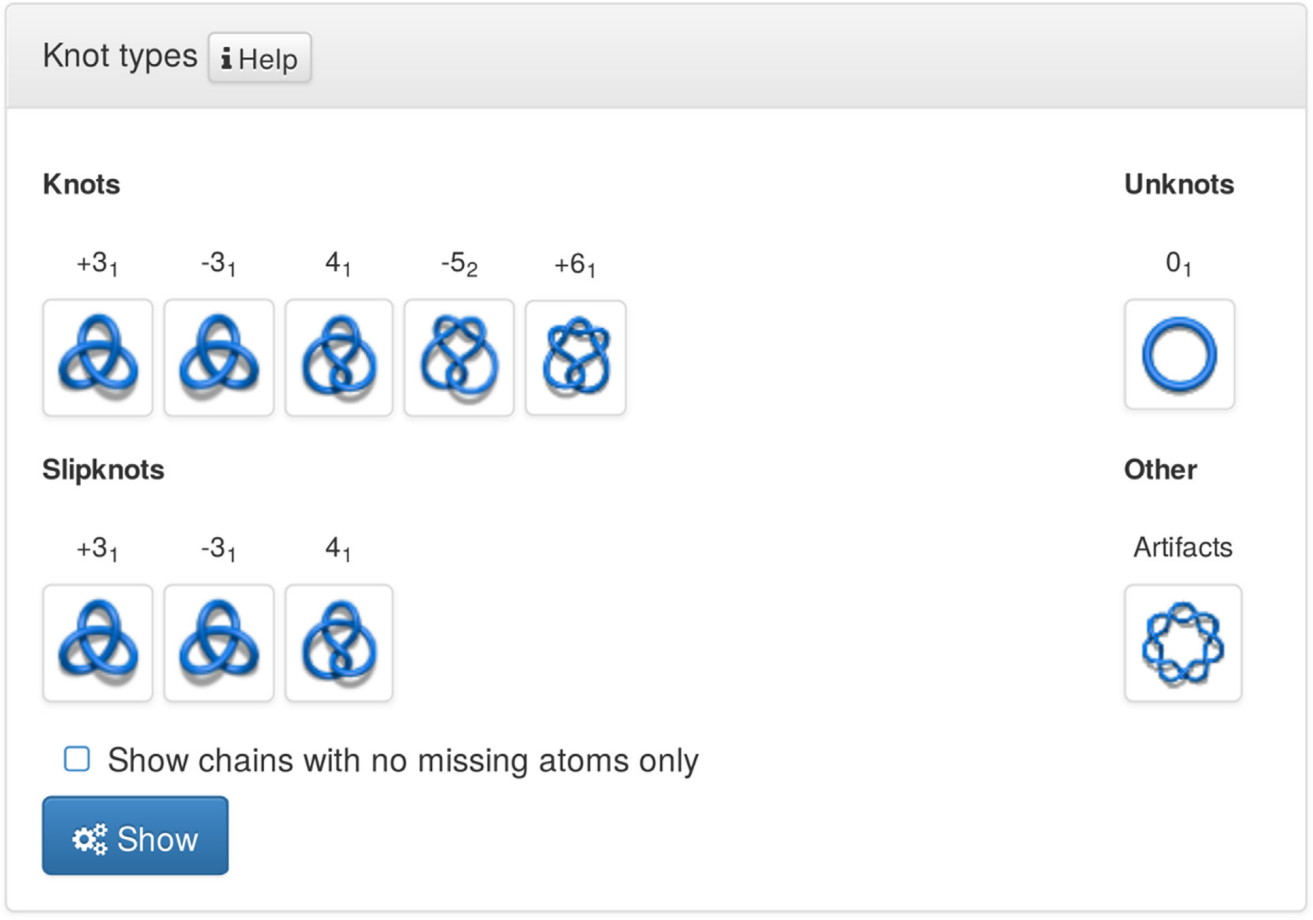
Searching the KnotProt database according to the knot or slipknot type. A schematic graphical representation is shown for each knot type, including its chirality. All structures in PDB that do not contain knots are listed under ‘Unknots.’ Structures for which a knot type is most likely improperly determined, e.g. due to missing atoms, are collected under ‘Artifacts.’

It is nontrivial to define the concept of knotting for an open chain. Only in the case of a closed chain is the knot type uniquely determined, and the techniques for classifying the knotting in open chains rely on closing the chain. However, for open chains the knot type can change depending on the way the chain is closed (26). To characterize the knotting specified by a rigid trajectory of an open chain and not by the particular way the chain is closed, one strategy is to pass from a deterministic to a probabilistic concept of knotting and ask a question: what is the most likely closed knot type specified by a given rigid trajectory of an open chain (25). To answer this question one might try to consider all possible closures of a given chain and analyze the frequency with which different knots result from such closures. Testing of all possible closures is practically impossible but there are closure methods that do not introduce a bias in the observed frequency of various knots. In the KnotProt database, we apply a random closure method, i.e. we connect protein endpoints several hundred times to two points randomly chosen from a set of vertices of the truncated icosahedron (i.e. a polyhedron representing, e.g. the geometry of C_60_ fullerene) positioned on a large sphere enclosing the analyzed chain. Subsequently these two points are connected by an arc lying on the surface of the sphere. The most frequently observed knot type for a given analyzed chain is then associated with that chain as its dominant knot type.

The knot types resulting from individual closures are determined by computing polynomial knot invariants. For quick computations of all analyzed subchains we use the Alexander polynomial. To detect chirality of the formed knots the HOMFLY polynomial is calculated for some of the analyzed subchains.

Computing knot polynomials is relatively fast for short chains, however it can be a very time consuming process for long chains (e.g. for proteins with more than 500 amino acids). Therefore, before computing the Alexander polynomial for a given chain (or fixed subchain), after closing terminals (for each random closure separately) we first reduce it to a shorter configuration using the KMT algorithm (27). This algorithm analyzes all triangles in a chain made by three consecutive amino acids, and removes the middle amino acid in case a given triangle is not intersected by any other segment of the chain. In effect, after a number of iterations, the initial chain is replaced by a (much) shorter chain of the same topological type, see the right panel in Figure 2.

### Knotting fingerprint and notation

The KnotProt database verifies not only if a given chain is knotted (or not), but it also analyzes all subchains of a given chain. This means that for a chain of length *N*, we analyze all subchains spanned between *C*_α_ atoms (amino acids) k and l (with 1 ≤ k < l ≤ *N*). For a given protein this information is presented in the database within the panel ‘Knotting data,’ in the form of an interactive ‘knotting fingerprint’ (matrix diagram), see top left panel in Figure 1. The knotting fingerprint was introduced in (1) and was motivated by (8). A knotting fingerprint is a triangular diagram where a point in a position (*k*,*l*) is colored according to the type of the knot detected for subchain (k,l). In particular, a slipknot corresponds to a configuration in which the whole chain is unknotted, but it has a knotted subchain, see Figure 3. On this triangular diagram the detailed locations of ‘knot core’, ‘knot tails,’ ‘slipknot loops’ and ‘slipknot tails’ are presented, see Figure 3 and definitions below. The same information is also shown in a structural (JSmol (28)) and sequential representation, appropriately colored. For various applications of knotting fingerprints see also (29).

Figure 3 (from (1)) presents examples of knotting fingerprints of a knot and two types of slipknots, and explains how their geometric properties are encoded in a matrix diagram. For a given chain of length *N*, all its subchains spanned between amino acids k and l (for 1 ≤ k < l ≤ *N*) are analyzed. If a subchain from k to l is knotted, then a point with coordinates (k,l) is denoted in the relevant color in a plot. For example, in the database, points representing knots 3_1_, 4_1_ and 5_2_ are respectively green, red and violet. The intensity of the color represents the percentage with which the given knot was detected, see Figure 1 or Figure 4.

In a given knot or slipknot, several geometric elements can be distinguished. These elements are denoted by different colors along the diagonal of a matrix diagram (see Figure 1 and schematic representation in Figure 3):

**- knot core** (thick line in Figure 3A, and in blue in KnotProt): the shortest subchain for which a knot is detected (i.e. after cutting an amino acid from any terminal of such a subchain, just a trivial knot would be detected); note that ‘knot core’ is defined also for a slipknot.
**- knot tail** (thin lines in Figure 3A, and in gray in KnotProt): a segment between one of the termini of a knotted chain and its ‘knot core’.
**- slipknot tail** (thin lines in Figure 3B and C, and in green in KnotProt): in a structure with a slipknot, the longest segment starting at one terminal, for which no change in topology is detected.
**- slipknot loop** (dashed lines in Figure 3B and C, and in orange in KnotProt): in a structure with a slipknot, a segment between a ‘slipknot tail’ and a ‘knot core.’

The knotting fingerprint takes into account missing atoms. In case this information is encoded in the PDB file, the ‘missing atoms’ are also listed in the ‘Chain information summary’; moreover, if these missing atoms overlap with the knot core, they are represented by gray strips as in Figure 4. In case the information about missing atoms is not encoded in the PDB file (i.e. there is no gap in the numbering of amino acids, but a distance between some pair of neighboring amino acids is substantially larger than 3.8 Å), red lines are shown in the matrix diagram. In both these cases the missing part of the chain is replaced by a line segment. This may affect the type of knot detected, so one should be careful in interpreting results in such cases. Missing atoms in the chain are denoted in sequence representation (bottom panel in Figure 1) as ‘-’.

#### Topological notation of knotted proteins

Frequently knotting fingerprints (matrices encoding the knotting types of all subchains) show the presence of more than one knot type or the same knot type appears in disjoint territories of the fingerprint. This feature was used to define a topological notation for knotted proteins. In this notation we list distinct knotted areas, ordering them according to the size of the subchain forming a given knot, starting with the largest one. For example the knotting fingerprint of chain A of *Escherichia coli* ketol-acid reductoisomerase shown in Figure 1 has the notation K4_1_3_1_ where K indicates that the entire protein is knotted and that it forms the 4_1_ knot but has a portion that forms 3_1_ knot. If the entire protein is unknotted but contains a slipknot, its topological notation starts with S. Among the complete protein structures deposited in the PDB, we found proteins with the following topological notations:

- knots: K3_1_, K4_1_3_1_, K4_1_, K4_1_4_1_, K5_2_3_1_3_1_, K6_1_6_1_4_1_3_1._
- slipknots: S3_1_, S3_1_3_1_, S3_1_3_1_3_1_, S3_1_3_1_3_1_3_1_, S3_1_4_1_3_1_, S3_1_4_1_3_1_3_1_3_1._

Interestingly, the topological notation is strongly conserved among ortologous proteins even if their structure highly diverged during hundreds of millions of years from their evolutionarily separation. Therefore, the topological notation can be used to identify proteins with the same or similar function, as a template to model new proteins (e.g. to impose topological constraints on threading), to identify new members of a given family, etc.

In KnotProt we also list (as ‘putative notations’) a few cases where the notation is associated to protein chains with undetermined fragments and where the ‘missing’ portion was replaced by a line segment. In such cases the line segment can pierce through the existing portion of the chain and introduce spatial structure that is an artifact. In particular, we found cases with topological notations K5_1_3_1_, S7_1_5_1_3_1_, K7_5_3_1_5_1_5_2_3_1_ or even K8_2_3_1_3_1_3_1_3_1_. At present we do not think that these notations reflect the notation of the entire protein structure of the respective proteins. Once complete structural information about these proteins is provided (e.g. by new crystallization results, or proper reconstruction), the KnotProt analysis will be repeated and the results will be updated.

### Proteins in the database

The data set taken for the current analysis by KnotProt consists of all 144 554 protein structures deposited in the PDB. We included non-X-ray entries and entries with *C*_α_-only entries. Those 144 554 chains were subsequently evaluated to take into account insertions in these sequences of all non-typical amino acids: MSE, FGL, LLP, SAC, SER, PCA, MEN, CSB, HTR, PTR, TYR, SCE, M3L, OCS, KCX, SEB, MLY, CSW, TPO, SEP, AYA, TRN. This analysis is performed so as not to introduce additional breaks along the protein chain. In the case of NMR structures, we took the first model with a given chain name obtained from the PDB server. This analysis gives the largest non-identical protein chain set. Out of those chains we identified around 1150 chains that possess either a knot or a slipknot. These currently comprise the KnotProt database.

### Database technicalities

Database website interface, communication mechanism between computational and remote servers, and parsers of remote files are written in the Python scripting language, with the Flask framework for dynamically generating HTML pages. Graphics (plots) are created with the matplotlib library, the structure view window is developed using JSmol (HTML5/JavaScript version). The service uses the SQLite 3 SQL database for data storage (user data as well as protein knots database). The web server uses apache2 with wsgi, and SGE queue for user jobs management.

Information about proteins is downloaded from the Protein Data Bank (PDB), directly from deposited XML files or by using RESTful services. CATH (30) data are downloaded a few times a year and is checked for new domain assignments for the KnotProt deposited entries. Pfam and EC data are fetched using the SIFTS service (31). The whole service is installed on standard linux boxes with 16–24GB of RAM, 12–24 CPU threads and CUDA-compatible coprocessors.

## DATABASE INTERFACE AND DATA PRESENTATION

### Single protein data presentation

After selecting a particular protein from the database (e.g. after browsing or searching the database, as described in the next section) users can view all information about it in the following screens:

**Knotting data**: The main part of this screen is shown in Figure 1; it contains the matrix diagram (knotting fingerprint) of a protein (top left), JSmol graphics representation of the protein (top right), a table listing all knots found in subchains of the protein (and detailed information about their lengths, depths, chirality, etc.) and the sequence representation of the protein with knot and slipknot elements (knot core, knot and slipknot tails and loops, etc.) highlighted in colors (bottom). The matrix diagram is interactive: after choosing a knot type (if more than one knot type is detected) from the table, the data corresponding to this knot is shown in the diagram. By default the data corresponding to the knot formed by the whole chain (for knotted proteins), or the most complicated slipknot (for proteins with slipknots) is shown in the diagram.
**Chain information summary**: this screen collects basic biological information about the protein: its size, molecule tags and keys, source organism, Enzyme Classification (EC), the number of missing residues, Pfam annotations, etc.; hyperlinks to the PDB, PubMed, Pfam and DOI (if available) are also included.
**Similar chains (by sequence)**: provides two lists: the first list contains the PDB codes of other chains deposited in the KnotProt database with at least 40% sequence similarity. The second list contains the PDB codes of either other chains of the homomultimeric complex with 100% sequence identity to the one deposited, or chains not yet processed by the KnotProt with at least 40% sequence similarity.
**Similar chains (by structure)**: lists PDB codes with the same super family or topology or homology, as defined by the CATH database.

### Browsing, searching and processing structures

There are three main options a user can choose from to view or analyze data:

1. ‘Browse database,’ which lists all structures currently deposited in the database; all these structures are also hyperlinked to other databases;
2. ‘Search database,’ which provides classifications of proteins with respect to their topological, biological, sequential and geometrical properties;
3. ‘Process my structure,’ which allows users to upload new polymer-like structures and analyze their topology, or analyze time evolution of entangled structures.

These options are summarized below and described on the website in a manual available under ‘Read more’. Apart from the above options a user can also search proteins according to their PDB code and chain notation.

#### Browse database

This option includes all protein chains deposited in the KnotProt database. They can be browsed in three different ways. The default screen presents a list of proteins with their PDB code (together with the chain number specified), the topological notation and the title used in the PDB header. Proteins with incomplete chains are denoted by an additional symbol of a broken chain element. Another option is to browse a list of names together with miniature figures of knotting fingerprints—this enables users to quickly identify some particular shape of matrix diagram. The third possibility is to browse a list of raw data, which is suitable for independent analysis. Upon choosing one of the listed proteins, the full information about it is presented as described in the section ‘Single protein data presentation’.

#### Search database

A user can search the database in various ways. In the default screen for this option (Knot type) the following sub-options can be chosen:

- ‘knot types’: contains four sub-classifications: Based on a type of knot, a type of slipknot, a list ‘Other’ of proteins with knots which we believe are artifacts (arising from broken chains), and a list ‘unknot’ of all unknotted (and not containing any knotted subchains) proteins in the PDB, see Figure 5.
- ‘fingerprint’: classification (separately for knots and slipknots) of proteins according to their topological notation (knotting fingerprint), such as K5_2_3_1_3_1_, S3_1_4_1_, etc.
- ‘knot length’ and ‘Knot/slipknot depth’: knots and slipknots are grouped according to the length of the knot core, as well as the length of the N-terminal and C-terminal tails; those lengths are used to classify knots as shallow or deep (for tails shorter or longer than 10 amino acids, respectively).

Moreover several other classifications can be chosen, according to: ‘Molecule keywords’, ‘Molecule tags’ (based on the classification from the PDB website), ‘PFAM family identifier’, ‘EC nomenclature’ (numerical classification for enzymes based on the chemical reactions they catalyze), ‘CATH classification’ (which includes class, architecture and topology) and ‘Keywords cloud.’ Based on these classifications, we have already found some new results (which are described below in the ‘Results’ section) and we believe that the database will provide the opportunity for other researchers to make many more new, deep discoveries.

#### Process my structure

A user can upload and analyze two types of data: either a single structure or a whole set of structures (e.g. a folding or unfolding trajectory). The data can be submitted either in PDB format or in a simplified ‘x-y-z’ format (containing only Cartesian coordinates of atoms, which enables users to analyze arbitrary polymers or open chains). In the case of a single structure, when knots or slipknots are detected by KnotProt, the relevant knotting fingerprint is constructed. In the case of a trajectory, an xtc format file (typical for Gromacs software) can also be uploaded (together with a gro or pdb file). To detect knotting in an open chain, a user can choose either our standard method or direct closure, i.e. connecting two termini by a line segment (for more details see section ‘Knot detection’). It is also possible to determine a knot type associated to any subchain of the whole chain—in this case a user provides the numbers of two atoms, which are then regarded as the beginning and the end of a subchain.

After completing the analysis a user can download pictures representing the protein topology (in SVG vector format, SVG rasterized, or PNG map) and the corresponding raw data in a simplified ‘x-y-z’ format. A structure uploaded and analyzed by a user is stored for 14 days (so that it can be viewed again).

## RESULTS

As of the fall of 2014, the KnotProt database contains data for 1150 identified proteins with knots or slipknots. These data reveal many facts previously unknown, which have been found using the various classifications provided by the database; some of these facts are summarized below. All data deposited and processed by KnotProt can be used to study biological, geometrical and physicochemical properties of proteins with non-trivial topology; examples of some applications are also listed below.

### Classification and new results

**Figure 6.**
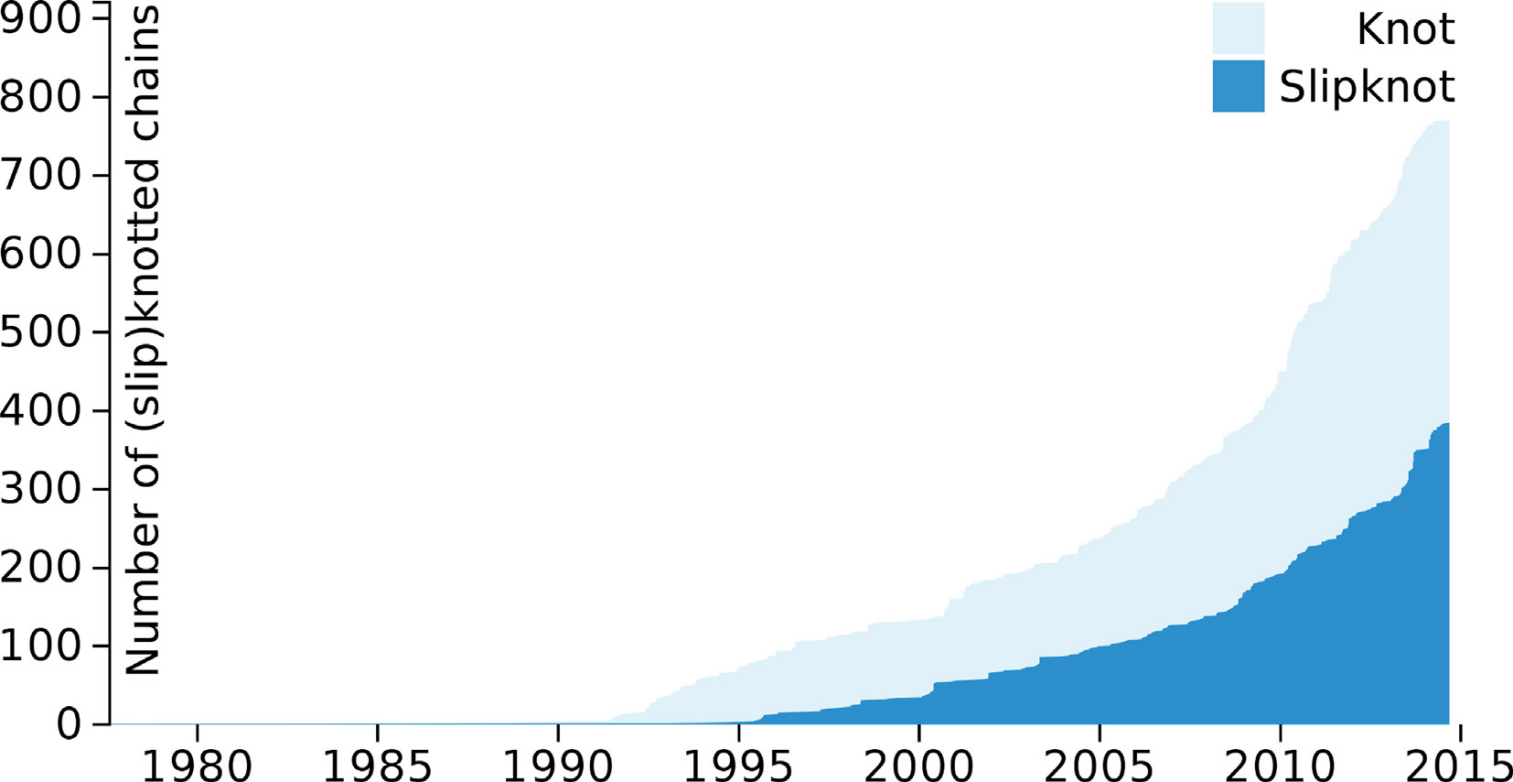
The number of proteins with knots and slipknots (from the KnotProt) contained in the PDB by year.

Many new results are summarized in classifications provided by the database. Here, we list some new results based on an initial inspection of the database. While some of the observations might be intuitive, many others require detailed analysis and should lead to new discoveries:

- there are few distinct topological notations: only 6 for proteins with knots (K3_1_, K4_1_, K4_1_3_1_, K4_1_4_1_, K5_2_3_1_3_1_, K6_1_6_1_4_1_3_1_) and six for proteins with slipknots (S3_1_, S3_1_3_1_, S3_1_3_1_3_1_, S3_1_3_1_3_1_3_1_, S3_1_4_1_3_1_, S3_1_4_1_3_1_3_1_3_1_); all knotted and slipknotted complete proteins in the PDB belong to one of these types (as of the fall 2014).
- there are substantially more knots than slipknots; in particular the most common type K3_1_ has been identified in 715 structures, while the most common slipknot type S3_1_ has been identified in 380 structures.
- the most typical knot length (in around one third of all knotted structures) is in the range 220–230 amino acids.
- knots are typically formed closer to the C-terminus of a protein.
- among molecular keywords, knots and slipknots appear most often in carbonic anhydrase (one third of all structures are of this type); other more common structures (involving around a few percent of all structures) include alkaline phosphatase, thymidine kinase and transporter, which are membrane proteins.
- knots and slipknots appear most for the molecule tags lyase, transferase and hydrolase.
- according to the PFAM family identifier, knots and slipknots arise most often in eucaryotic-type carbonic anhydrase.
- according to the Enzyme Classification, the carbonate dehydratase (EC 4.2.1.1) is the most common enzyme type of knotted proteins (537 occurences in KnotProt) and alkaline phosphatase (EC 3.1.3.1) is the most common slipknot (65 occurrences in KnotProt).
- α-β is the most common CATH class; ‘roll’ (482 cases) and ‘3-layer(αβα) sandwich’ (244 cases) are the most common CATH architectures for which knots or slipknots appear; ‘carbonic anhydrase II’ is the most common CATH topology.
- the number of knotted and slipknotted proteins (from KnotProt) in the PDB over the years is shown in Figure 6.

### Possible applications

A lot of information can be deduced from the results of trajectory analysis. For example, users can enter momentary configurations of proteins during simulated folding to detect, for example, when the knot formation is initiated and how the position and size of the knotted core evolves during protein folding. The results of the trajectory analysis also can be used to characterize the probability of knot occurrence in various reaction coordinates, such as native contacts (the number of contacts existing in protein in the native state) or RMSD, as discussed in (1). As another application, one can analyze knots and slipknots from the viewpoint of thermodynamics processes, as discussed in (32).

## DISCUSSION

The KnotProt database contains various information about proteins in the PDB whose backbone chains form knots or slipknots. Currently there are around 1150 such chains. These data are automatically updated every week, immediately after new structures are deposited in the PDB. One of the main features of the KnotProt database is the presentation of precise topological information for every knotted and slipknotted protein encoded in the form of the knotting fingerprint. We further present the topological notation of the proteins, the knot and slipknot lengths and depths, the locations of the knot cores and slipknot loops, etc. These data enable users, for example, to find correlations between the locations of active sites and special points determined by the knotting fingerprint—interestingly, recent work shows that active sites are typically located inside the knotted core (1). Identification of those properties is critical, e.g. for drug research, or *de novo* and experimental determination of new proteins. It should be noted that, according to the classifications provided by KnotProt, the knotting fingerprint is a highly conserved feature of a protein. For example, in a family of membrane transporters, the sequence homology is as low as 6%, although all members of this family possess a similar knotting fingerprint. The possibility to analyze in KnotProt any new structure (and trajectory) uploaded by a user is an additional useful feature.

The KnotProt database also includes many other important biological and geometrical characteristics. Those features are easily accessible and related to the knotting fingerprint. They can be used to find a set of proteins with a given biological activity or with a particular type of a knot. The classification based on sequence similarity allows users to find all homological proteins, the Pfam classification allows users to identify all members from given family and the Enzyme Classification enables users to identify enzymatic roles of proteins with knots and slipknots. It is hard to overestimate the importance of providing data from various classifications—all this information gives significant new insights into the structure and origin of topologically nontrivial proteins, and could lead to many new experiments.

### Comparison to other web servers

We are aware of two other web servers: ‘Protein knot server’ (33) and ‘pKNOT v.2’ (34), which allow users to determine whether an uploaded protein is knotted. These web servers are substantially different from the KnotProt database; in particular the KnotProt database provides many more options and information. Both web servers (33) and (34) can only check if a given protein chain is knotted; in particular they cannot detect slipknots. However the KnotProt database analyzes all subchains of a protein chain, can detect slipknots and determine the knot notation and construct the knotting fingerprint in the form of an interactive matrix diagram (which shows the location of a knotted core, knot and slipknot tails and loops, etc.). The KnotProt database also summarizes valuable information and classifications described above, which are not available in any other internet resources. Moreover, in KnotProt the user can analyze the topology of any new structure (or the whole trajectory), uploaded in one of a few convenient data formats. In addition the KnotProt database is automatically updated. We are also aware of the PyKnot plugin (35) and the Rknot package (36) for visualization and characterization of knots in proteins; however, these programs do not detect slipknots, do not construct knotting fingerprints and do not provide various classifications of entangled proteins, which are the main features of the KnotProt database.
